# Aqueous humour outflow improvement after excimer laser trabeculostomy

**DOI:** 10.1136/bjo-2025-328720

**Published:** 2026-02-18

**Authors:** Martin Kallab, Isabella Vieboeck, Parsa Panahi, Kaweh Mansouri, Matthias Bolz, Alex S Huang, Clemens Strohmaier

**Affiliations:** 1 Ophthalmology/Optometry, Johannes Kepler Universitat, Linz, Austria; 2 Glaukoma Unit, Swiss Visio Montchoisi, Lausanne, Switzerland; 3 Department of Ophthalmology, University of California San Diego, La Jolla, California, USA

**Keywords:** Aqueous humour, Glaucoma, Treatment Surgery, Physiology

## Abstract

The purpose of the study was to investigate changes in aqueous humour outflow (AHO) after the Elios glaucoma procedure (excimer laser trabeculostomy, ELT) using sequential AHO angiography. AHO angiography using two different tracers was performed in six eyes of five patients undergoing cataract surgery combined with ELT. AHO was compared in regions of interest in the nasal quadrant.

AHO improved significantly after ELT (p=0.03) overall, and each patient showed individual, qualitative AHO improvement. Interestingly, AHO improvement extended beyond the site of ELT application. Further studies are needed to investigate the correlation of AHO improvement and clinical efficacy.

## Introduction

 Minimally invasive glaucoma surgery procedures (MIGSs) are an advancement in glaucoma care, as they potentially provide surgical intraocular pressure (IOP) lowering with a favourable risk profile compared with filtration surgery procedures.

Among available MIGS approaches, bypassing the trabecular meshwork (TM) using either stent implantation or surgical ablation/removal is the most common.^1^ These procedures aim to reduce IOP by addressing the proximal conventional outflow system, which is altered in glaucoma.^2^ However, the success rates of these procedures show variability between patients, sometimes eyes, but most notably between in vivo and ex vivo reports.^3 4^

The reasons for inconsistent MIGS results are poorly understood, and reports on the effects of TM bypass on aqueous humour outflow (AHO) physiology are scarce. TM bypass by two iStents led to AHO improvement and/or could result in new AHO pathways in one study.^5^ A case report of AHO improvement after bent-angled needle goniotomy (BANG) found qualitative improvement of AHO at the surgical site.^6^ Recent laboratory work has demonstrated the potential of angiography-guided TM bypass,^7^ but quantification techniques for clinical follow-up studies are currently missing.

The purpose of the present study was to study and quantify AHO improvement after excimer laser trabeculostomy (ELT) using the Elios procedure.

## Methods

AHO outflow patterns were acquired in six eyes of five patients undergoing routine cataract surgery and ELT (Elios Vision, Germering, Germany). Table 1 provides clinical patient characteristics. Routine cataract surgery was performed by one experienced surgeon (CS). Only cohesive viscoelastics were used (Healon Pro, Johnson & Johnson Vision, Vienna, Austria).

**Table 1:** Patients characteristics before and 3 months after surgery

	Mean±SD	Range
Age	71.33±12.00.1	57–85
Gender	4 female, 2 male	
Diagnosis	4 POAG, 2 OHT, 1 PACG	
VF MD (dB)	−1.19±1.38	−3.73 to 0.11
RNFL thickness (µm)	89±15.66	63–105
IOP baseline (mm Hg)	19±5.22	13–24
IOP 3 months (mm Hg)	13.33±2.42	10–16
Meds baseline	2±1.1	1–3
Meds 3 months	1.17±1.47	0–3

IOP, intraocular pressure; Meds, number of IOP lowering medication classes; OHT, ocular hypertension; PACG, primary angle closure glaucoma; POAG, primary open angle glaucoma; RNFL, retinal nerve fibre layer; VF MD, visual field mean deviation.

Following cataract surgery, AHO angiography was performed before and after the ELT procedure. Two anterior chamber (AC) maintainers (Self Retaining ACM (Lewicky)) were used to facilitate fluid exchange, with one for inflow and one for outflow. Pharmaceutical-grade indocyanine green and fluorescein were diluted to 0.4% and 2%, respectively. AHO angiography using ICG was performed at baseline (before ELT) and fluorescein after the ELT procedure. AHO patterns were recorded using a digital angiography system suitable for the operating room (Spectralis FLEX, Heidelberg Engineering, Heidelberg, Germany) at 15, 30, 45, 60 as well as 90 s after AC fluid exchange with the respective tracer.

The ELT procedure was performed according to the manufacturer’s recommendations, placing 10 laser spots in the nasal quadrant using a temporal surgical approach.

**Figure 1:**
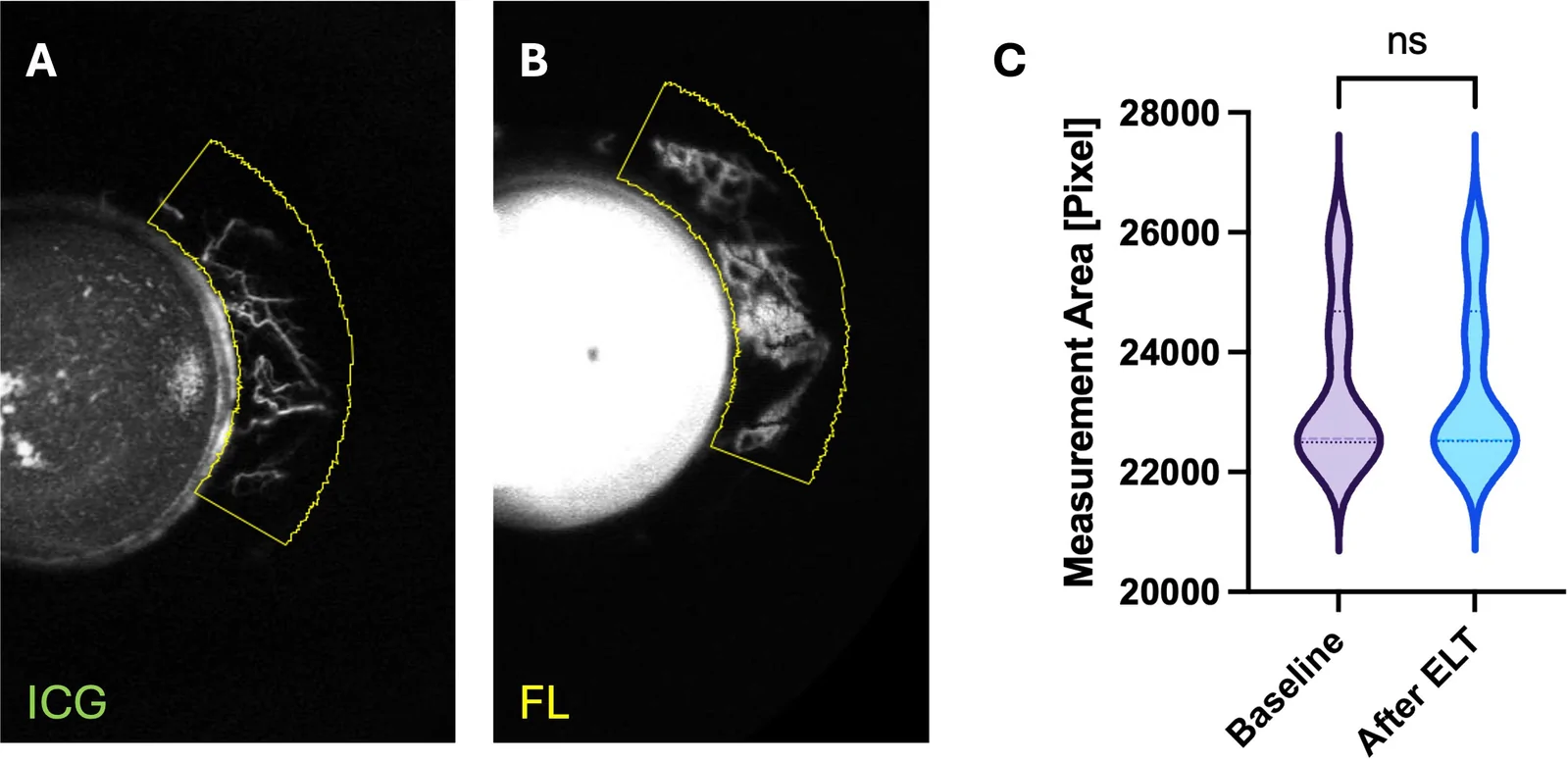
Image analysis procedure. (A, B) Highlight the region of interest (ROI—in yellow) for ICG angiography (**A**) and fluorescein angiography (**B**) images. Within the ROI, the average brightness was analysed to quantify AHO. (**C**) Compares the measurement areas between the two sequential angiography acquisitions. No significant difference in the measurement area between the sequential angiographies was found (C, p=0.98). AHO, aqueous humour outflow; ELT, excimer laser trabeculostomy; FL, fluorescein angiography; ICG, indocyanine green angiography; ns, not significant.

After the surgical procedures, further processing was done in ImageJ. Images acquired after 60 s were used for analysis. Figure 1 illustrates the image analysis process. A standardised region of interest (ROI) was placed in the nasal segment of the images to cover the target region of the ELT application (figure 1A,B), and the mean brightness of the ROI was analysed. The two ACMs served as a reference point for the ROI placement (as the camera system had to be removed and repositioned between the angiography captures to perform ELT). The statistical analysis compared the average signal intensity (image brightness) in the ROI using paired t-tests (Prism GraphPad V.10, GraphPad Software, Boston, USA). Since the ROI had to be positioned individually to compensate for image rotation, the analysed area was compared as well (figure 1C).

## Results

**Figure 2:**
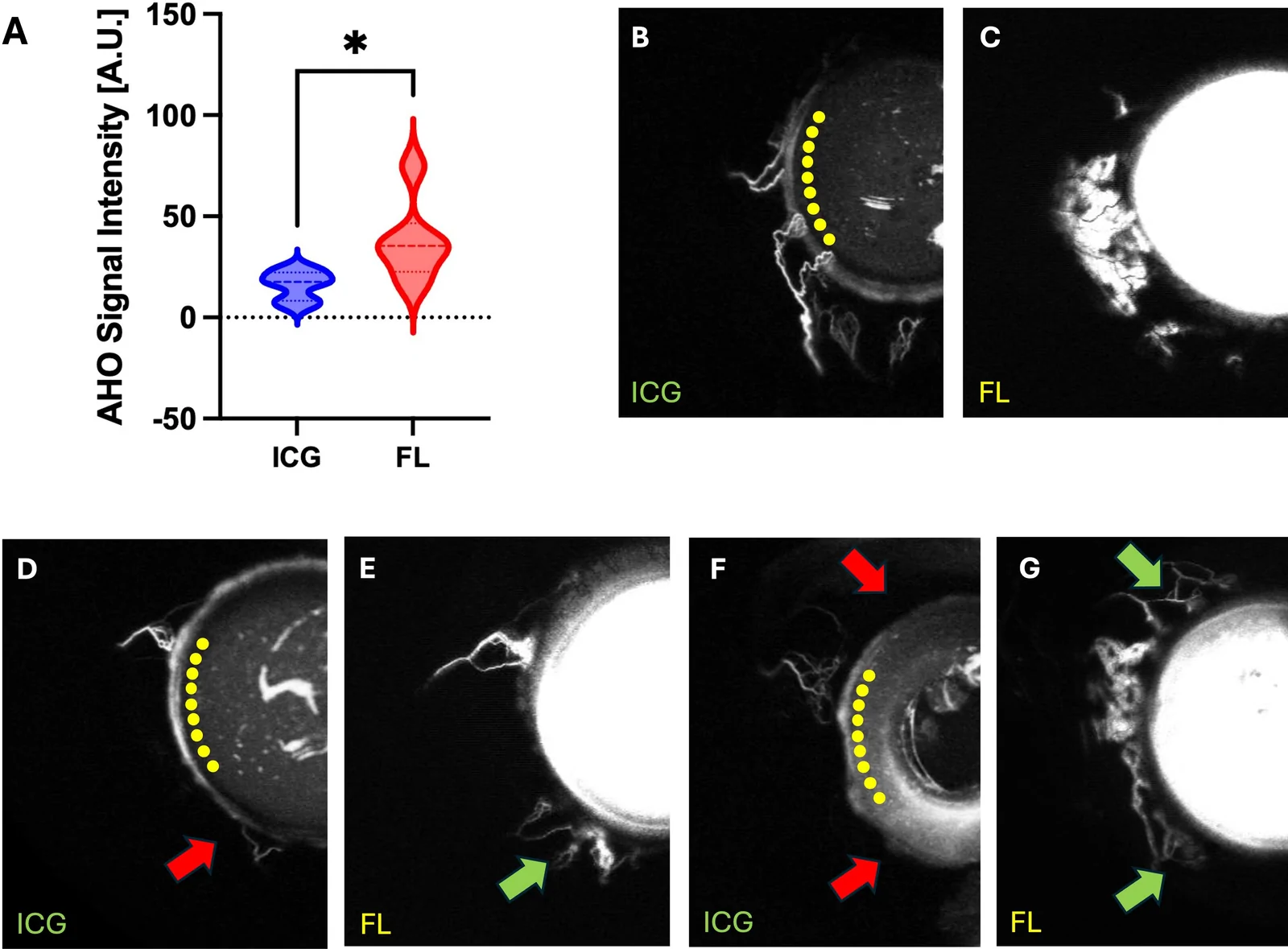
(**A**) AHO signal intensity before and after the ELT procedure. Performing ELT in the nasal quadrant led to a significant increase in AHO (p=0.03). (**B–G**) Representative AHO angiography examples exhibiting different patterns of AHO improvement. The yellow dots mark the surgical site at which 10 ELT spots were applied. In (**C**), the AHO improvement was located in the region of laser application. In (**E, G**), AHO improved beyond the surgical site. The red arrows in (**D, F**) highlight regions without an AHO signal in the baseline angiography. After the ELT procedure, those regions exhibited AHO (green arrows). * = p< 0.05. AHO, aqueous humour outflow; ELT, excimer laser trabeculostomy; FL, fluorescein angiography; ICG, indocyanine green angiography.

After uncomplicated cataract surgery, AHO angiography with ICG and fluorescein could be acquired in all patients. As reported previously, AHO at baseline showed a segmental pattern, with high-flow and low-flow regions of variable length adjacent to each other. Nasally performed ELT surgery (irrespective of segmental patterns) significantly increased AHO (p=0.03, figure 2A). The distribution pattern of AHO improvement, however, was variable. In some patients, the AHO improvement pattern correlated spatially with the ELT treatment (figure 2B,C), while in others, AHO improvement was seen in segments adjacent to the treatment site (figure 2D–G).

## Discussion

The present study demonstrates AHO improvement after the Elios ELT procedure and suggests an analysis protocol to quantify this change. Previous reports of AHO in patients have demonstrated AHO improvement after iStent implantation as well as BANG.^5 6^ It should be noted that the former study performed sequential AHO angiographies before cataract surgery and after the TM intervention. In theory, our approach should isolate the effect of the TM bypass, but the actual significance of this methodical alteration is currently unknown.

Studying AHO in glaucomatous eyes and before/after glaucoma surgeries is important, as the conventional outflow pathway is altered in glaucoma, and the only currently available parameter to assess the efficacy of an intervention is the IOP effect. Importantly, IOP is the summation of multiple (and potentially unknown) variables that may influence the overall outcome. One of those factors is the spatial placement of TM bypass surgery. AHO is known to be segmental, that is, exhibiting high-flow and low-flow regions when investigated with different tracers.^8^ Recent laboratory work has demonstrated that those segments are related to differences in outflow facility or resistance, and selective bypass of low-flow regions yields a larger treatment effect.^7 9^ Follow-up studies to confirm this finding in vivo are necessary, but therefore, standardised imaging and analysis protocols need to be in place.

A finding of the present study is that AHO improvement is not confined to the surgical site per se but can extend to adjacent regions. This might be expected, if Schlemm’s canal is regarded as a circular tubular structure between the TM and collector channels. Recent evidence, however, challenged this concept, as valve-like structures in Schlemm’s canal could be demonstrated.^10^ A non-uniform Schlemm’s canal could restrict the accessibility of collector channels after TM bypass, thereby limiting the AHO improvement achievable.

While the finding of AHO improvement beyond the surgical site is novel, it should be interpreted with caution, as the current study is of relatively small size and was not intended to study this phenomenon. Furthermore, the use of tracers with different molecular weight might cause artefacts in the observed AHO signal.

In summary, the present study demonstrates AHO improvement by the Elios procedure and suggests a standardised protocol to quantify those changes. AHO improvement beyond the surgical site is a novel finding that warrants further study.

## Data Availability

Data are available on reasonable request.
